# Multiple drug resistance and biocide resistance in *Escherichia coli* environmental isolates from hospital and household settings

**DOI:** 10.1186/s13756-018-0339-8

**Published:** 2018-04-02

**Authors:** Bothyna Ghanem, Randa Nayef Haddadin

**Affiliations:** Department of Pharmaceutics and Pharmaceutical Technology, School of Pharmacy, The University of Jordan, Amman, 11942 Jordan

**Keywords:** *Escherichia coli*, Biocide, Environment, ESBL, Hospital, Multiple drug resistance

## Abstract

**Background:**

Antibiotic resistance of environmental *Escherichia coli* in hospitals could be increased due to extensive use of biocides resulting in serious infections. In this study, the prevalence of antibiotic resistance of environmental isolates of *E. coli* from hospitals and household settings were evaluated and compared. In addition, the association between biocide minimum inhibitory concentration (MIC) and multiple drug resistance (MDR) was investigated.

**Methods:**

Environmental samples were collected from different homes and hospitals in Amman, Jordan. The isolates were identified phenotypically and by PCR. Antibiotic susceptibility tests and MIC of selected biocides were performed on the isolates. Screening for *bla*CTX-M group 1 was also performed.

**Results:**

Of 21 *E. coli* strains isolated, 47.6% were MDR and 67.9% were phenotypically identified as extended spectrum beta-lactamase (ESBL) producers. The occurrence of these ESBL isolates was comparable between household and hospital settings (*P* > 0.05). The MIC values of the biocides tested against all isolates were well below the in-use concentration of biocides. Moreover, the MICs of biocides were comparable between isolates from households and those from hospitals (*P* > 0.05). No association was found between MDR and biocide MIC (P > 0.05). Most of ESBL isolates harboured *bla*CTX-M 1.

**Conclusions:**

The extensive use of biocides in hospitals is not associated with MDR nor does it affect the MIC of biocides against *E.coli*.

## Background

Health care associated infections (HAIs) are known to contribute to morbidity and mortality among patients affected by them. In addition, they cause significant medical and financial consequences accompanied by emotional devastation [1]. Among the factors contributing to increased risk of HAIS are poor facilities cleaning and inadequate disinfection of health care settings [1]. These issues have led to the extensive use of biocides (including antiseptics and disinfectants) in hospitals. Since biocides have some common properties with antibiotics regarding their activity, mechanism of action and development of resistance [2], there is a possibility that resistance to biocides can contribute to resistance to antibiotics [3]. However, the contribution of biocide use to antibiotic resistance is still controversial and, despite some evidence, remains largely unproven. Several studies have shown that certain disinfectants have increased the expression of specific multiple drug resistant (MDR) efflux pumps which eventually resulted in resistance to some antibiotics [4]. Other studies have shown that over-exposure of bacteria to disinfectants results in reduced susceptibility towards some antibiotics [2, 5]. On the other hand, other studies have failed to show any cross-resistance between biocides and antibiotics [6, 7].

*E. coli* is a highly diverse species with respect to its virulence and pathogenicity. It is widely distributed in open systems and can easily spread in the environment causing risks to human health [8]. *E. coli* is one of the most common bacteria causing nosocomial infections. Its presence on inanimate surfaces in hospitals is one of the major infection control challenges facing hospitals [9]. In fact, contaminated surfaces and inanimate objects (fomites) are considered reservoirs for pathogen transmission to the patients. Therefore, the use of biocides in hospitals is of a paramount importance to control infections and transmission of pathogens.

In this study the biocides were selected to represent different chemical classes that are used extensively in Jordan in hospital and/ or household settings. Ethanol is an alcohol, which is used as antiseptic in hospitals and community settings. 4-Chloro-3,5-xylenol (known as chloroxylenol) is a phenolic compound that is used as a general antiseptic and disinfectant in the community. Iodine is a halogen, which is used in the form of povidone-iodine as a preoperative antiseptic in hospitals and for wound disinfection in household and hospital settings. Cetrimide is a member of the quaternary ammonium compounds that are incorporated in many biocidal preparations in combination with other biocides that are used in hospitals as antiseptics or disinfectants.

The aim of this research is to evaluate and compare the prevalence of antibiotic resistance in *E. coli* isolated from two distinct environments; hospital settings, where biocides are extensively used, and household settings, where biocide use is limited. *E. coli* was selected since it is recognized as an indicator for the presence of other Enterobacteriaceae and is a common cause of nosocomial infections. The collected isolates were assessed for the presence of potential association between antibiotic resistance and resistance to biocides, which was measured by an increase in their minimum inhibitory concentration (MIC).

## Materials and methods

### Sample collection

The environmental samples included in the study were collected from two hospitals, Prince Hamzeh (PH) Hospital and Jordan University (JU) Hospital and ten resident homes located in Amman-Jordan. Sample collection was performed from March to October 2016. The samples were collected using sterile swabs (Amies Transport media, Max Protect, China) that were pre-moistened with Amies medium present in the tube. The swab was rolled and moved over the surface to be sampled. After sampling, the swabs were transferred to the laboratory within one to two hours to be processed. In the laboratory, the swabs were cut off aseptically and placed in Lauryl sulphate tryptose broth (LSB) for enrichment and incubated overnight at 35^o^ ± 2^o^Ċ.

The two hospitals included in the study are among the largest in Amman. Each hospital treats on average more than 500,000 patients annually. Ethical permission to undertake sampling was obtained from both hospitals. Biocides used within the hospitals were recorded. The two hospitals use ethanol based gels and solutions as antiseptic for healthcare personnel, pre-injection disinfection and for visitor use. Different quaternary ammonium compounds based products are used for general disinfection and antisepsis within the two hospitals. Iodine in the form of povidone-iodine is used for wound disinfection and preoperative skin treatment. The hospitals apply strict disinfection policies that are monitored by infection control teams. Samples from hospital environments were collected from the floors, elevators, curtains, patient beds, windows, door knobs, nursing cabinets, bathroom sinks, drains, pressure devices, magnetic resonance device, operation equipment, dialysis device, trolleys, and any device that is transferrable among patients and medical staff. The samples were collected from different hospital wards. In total 344 swab samples were collected from both hospitals.

The homes included in the study apply routine cleaning to the premises using detergents. These homes occasionally use hypochlorite (a halogen) or chloroxylenol (e.g. Dettol®) based preparations for general disinfection. Povidone iodine and ethanol (70%) were the most common antiseptics used in these homes to treat bruises or cuts if occurred. Samples from household environment were collected from the floors, door knobs, bathroom sinks and kitchen sinks. The number of samples collected from the 10 homes was 86.

### *E. coli* Isolation and identification

A loopful of LSB culture was streaked on MacConkey agar medium and incubated overnight at 35^o^ ± 2^o^Ċ. Morphologically distinctive pink colonies were isolated and identified biochemically for oxidase production, Kligler’s iron agar, urease, gas, indole production and then identified using API 20 E kit (Biomerieux, France). The potential *E. coli* isolates were confirmed genetically using PCR method.

### Antibiotic susceptibility test

Antibiotic susceptibility test was performed using disc diffusion test according to Clinical and Laboratory Standard Institute (CLSI, 2016) guideline [10] using the following antibiotics: Amoxicillin, Amoxicillin-clavulanic acid, Cefaclor, Cefixime, Nitrofurantoin, Cefuroxime, Amikacin, Ciprofloxacin, Imipenem, Trimethoprim-Sulfamethoxazole, Doxycycline. These antibiotics represent the major antibiotic classes which have known activity against *E. coli* and are used clinically. *E. coli* ATCC 25922 was used as a quality control strain to validate the method [10].

The isolates were further tested to phenotypically detect extended spectrum beta lactamase (ESBL) producing bacteria using double disc diffusion test [10]. In this test, cefotaxime and ceftazidime discs alone and in combination of clavulanate were used. The isolate is considered ESBL producing if there is ≥5-mm increase in the zone diameter for antibiotic tested in combination with clavulanate vs the zone diameter of the antibiotic when tested alone [10].

### MIC determination of biocides

Minimum inhibitory concentrations of ethanol, chloroxylenol, cetrimide and iodine were determined using a broth microdilution method according to CLSI, but with slight modification. Stock solutions of ethanol, cetrimide and chloroxylenol were prepared in Mueller Hinton broth (MHB) to get final concentration of 200 mg/ml, 400 μg/ml and 300 μg/ml respectively. In order to enhance the solubility of chloroxylenol in MHB, it was dissolved first in dimethylsulfoxide (DMSO) and the final volume completed by the addition of MHB. The lowest concentration of DMSO needed to ensure complete solubility of chloroxyleneol in MHB was 5%. To ensure that DMSO at 5% concentration has no inhibitory effect on the isolates, positive control containing 5% DMSO in MHB was prepared for each isolate. Since MHB medium contains starch as an ingredient, TSB was used for MIC determination of iodine. Iodine was solubilized with potassium iodide at 1:2 ratio (I_2_: KI) in TSB to get a stock solution of iodine (1300 μg/ml). Aliquots (200 μl) of each stock solution were dispensed into the wells of a microtitre plate. Double serial dilutions were performed using broth. Each trial was performed in five replicates. Aliquots (20 μl) of each bacterial culture adjusted to 5 × 10^6^ CFU/ ml were used to inoculate the microtitre plate wells to yield a final concentration of ca 5 × 10^5^ CFU/ ml. The microtitre plate was incubated for 20 h at 35^o^ ± 2^o^Ċ. MIC was determined by visual inspection. For ethanol, since the difference between consecutive concentrations is large, linear serial dilutions were performed after determining its MIC by double serial dilutions. *E. coli* Nissle 1917 was used as a control for MIC testing. This strain is a kind gift from Ardeypharm GmbH, Germany. It is a probiotic non-pathogenic microorganism utilized clinically to treat many gastrointestinal disorders including diarrhoea, ulcerative colitis and uncomplicated diverticular disease [11].

### DNA extraction

The Wizard® Genomic DNA Purification Kit (Promega, England) was used to isolate DNA from the isolated *E. coli* strains. The kit was used according to manufacturer’s instructions.

### PCR primers and conditions

*bla*CTX-M group 1 gene and *E. coli*16s *rRNA* gene: PCR reaction was performed using 3 μl of the extracted DNA (2 μl for *E. coli* 16S *rRNA* gene), and 0.4 μM of each of the *bla*CTX-M group 1 gene forward and reverse primer and the (16 E1, 16 E2 and 16 E3) primers of *E. coli* 16S *rRNA* gene (Table 1). The gene was amplified using 12.5 μl of PCR Master Mix 2× (GoTaq® Green Master Mix, Promega, USA). The volume was made up to 25 μl using nuclease free water. Cycling conditions for *E. coli* 16S *rRNA* gene were applied according to Tsen et al., [12]. Cycling conditions for *bla*CTX-M 1 gene were applied according to Mirzaee et al., [13]. The amplified gene products were analyzed using 2% agarose gel electrophoresis and visualized by (UVP) system (Alpha Imager®, Japan) using Redsafe™ (Intron biotechnology, Korea).

**Table 1:** The targeted genes, primer sequence and product size

Target	Detection primer	Primer (sequence 5′ to 3′)	Product size (bp)	Reference
*E. coli* 16S rRNA	16 E1 (F)	GGGAGTAAAGTTAATACCTTTGCTC	584	12
	16 E2 (R)	TTCCCGAAGGCACATTCT		
	16 E3 (R)	TTCCCGAAGGCACCAATC		
*bla*CTX-M group 1	CTX-M-7 (F)	GCGTGATACCACTTAACCTC	260	13
	CTX-M-8 (R)	TGAAGTAAGTGACCAGAATC		

### Statistical analysis

The results were statistically analysed using the non-parametric Mann Whitney U test and the Chi square test as relevant. Analyses at 95% confidence level were performed. The analysis was performed using IBM SPSS Statistics version 23.

## Results

The total number of samples collected was 430; 175 samples were taken from PH hospital, 169 samples from JU hospital and 86 samples from household settings. The potential number of *E. coli* isolates identified by API kit was 21. These isolates were confirmed to be *E. coli* by PCR followed by agarose gel electrophoresis. Accordingly, the prevalence rate of *E. coli* in all the settings was 4.9%; seven isolates from PH hospital, four isolates from JU hospital and 10 isolates from households (Table 2).

**Table 2:** The antibiogram of *E coli* isolates from the environment of the two hospitals and 10 homes

Facility	Isolate	Amx	Amc	Cfc	Cfu	Cfx	Ctz	Ctx	Imp	Nit	Amk	Cip	Tms	Dox	MDR
Household settings	A126	R	S	R	R	R	R	R	S	S	S	S	S	S	No
	A409-c	I	I	R	R	R	R	R	S	S	S	S	R	I	No
	A410-a	R	R	S	S	S	R	R	S	S	S	S	R	S	Yes
	A410-b	R	R	R	R	R	R	R	S	I	R	S	R	S	Yes
	A411	R	S	R	R	R	R	R	S	S	S	S	R	I	Yes
	A413	R	R	R	R	R	R	R	S	S	S	S	R	S	Yes
	A414	R	S	R	R	R	S	S	S	S	S	R	R	S	Yes
	A801	R	I	S	I	S	S	S	S	S	S	S	R	I	No
	A814-a	I	S	S	I	S	S	S	S	S	S	S	S	S	No
	A824-a	S	S	S	S	S	S	S	S	S	S	S	S	S	No
PH Hospital	B107-a	R	S	R	R	R	S	S	S	R	S	R	R	R	Yes
	B425-a	S	S	R	R	R	R	R	S	S	S	S	R	I	No
	B425-b	S	S	R	R	R	S	R	S	S	S	S	R	S	No
	B426-a	R	R	R	R	R	R	I	S	S	S	R	R	R	Yes
	B475-a	R	I	R	R	R	R	R	S	S	S	R	R	R	Yes
	B506-a	S	S	S	S	S	S	S	S	S	S	S	R	R	No
	B705-b	R	S	R	R	R	R	R	S	S	S	S	R	S	Yes
JU Hospital	C604	R	S	S	I	S	S	S	S	S	S	S	R	I	No
	C705	S	S	R	I	S	R	R	S	S	S	S	S	S	No
	C715-b	I	S	S	I	S	S	S	S	S	S	S	S	S	No
	C906	R	S	R	R	R	R	R	S	S	S	R	S	I	Yes
	E. coli ATCC	S	S	S	S	S	S	S	S	S	S	S	S	S	No

Amx: amoxicillin, Amc: amoxicillin-clavulanate, Cfc: cefaclor, Cfu: cefuroxime, Cfx: cefixime, Ctz: ceftazidime, Ctx: cefotaxime, Imp: imipenem, Nit: nitrofurantoin, Amk: amikacin, Cip: ciprofloxacin, Tms: trimethoprim-sulfamethoxazole, Dox: doxycycline. MDR: multiple drug resistant

**Fig. 1:**
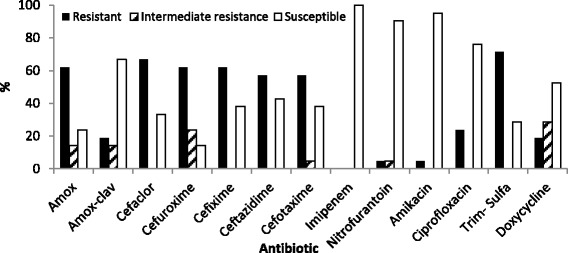
Percentage of resistant, intermediate resistant and susceptible *E. coli* strains versus the antibiotics tested

Antibiotic susceptibility tests have shown that the majority of the isolates (71.4%) exhibited resistance to Trimethoprim-sulfamethoxazole (Fig. 1). Resistance to amoxicillin was seen in 61.9% of the isolates. When clavulanic acid was combined with amoxicillin (amoxicillin-clavulanic acid), the resistance was seen in only 19% of the isolates. Resistance to cephalosporins (second and third generation) was considerable and ranged from 57% to 66.7% of the isolates. However, no resistance was detected against imipenem. Multiple drug resistance (MDR), that is resistance to three antibiotics or more from different classes, was observed in 10 isolates from the 21 *E. coli* strains (47.6%, Table 2). MDR *E. coli* isolates were found to comprise 50% (5 isolates), 57.1% (4 isolates) and 25% (1 isolate) of the isolated *E. coli* strains from households, PH hospital and JU hospital respectively (Table 2). The prevalence of MDR *E. coli* in the household environment was comparable to that in hospitals (*p* = 0.59). Moreover, 13 strains out of 21 were phenotypically identified as ESBL-producers (61.9%). The prevalence of the phenotypically identified ESBL *E. coli* in households was 60% and in hospital settings were 71.4% and 50% for PH & JU Hospital respectively. *bla*CTXM group 1 was found in 11 strains (52%) out of the 21 strains.

The results of biocides MIC against *E. coli* isolates are given in Table 3. All MIC values measured are well below the in-use concentrations of these biocides (Table 3). It is noteworthy that the quality control strain (ATCC 25922) and the probiotic strain (Nissle 1917) have MIC values that overlap with MIC values of hospital and household isolates. Moreover, the MIC values of the isolates collected from hospitals were found to be comparable to those collected from households (*p* = 0.23).

**Table 3:** MIC values of the biocides tested (mg/ ml) against different isolates compared to the in-use concentration (mg/ ml). Each result is the average of five replicates ± SD

Environment	Strain NO.	Cetrimide MIC±SD	Chloroxylenol MIC±SD	Ethanol MIC±SD	Iodine MIC±SD
Household settings	A126	0.2 ± 0	0.1 ± 0	55 ± 12	0.4 ± 0.2
	A409-c	0.2 ± 0	0.2 ± 0	75 ± 0	0.4 ± 0.1
	A410-a	0.4 ± 0.1	0.2 ± 0	55 ± 12	0.4 ± 0.2
	A410-b	0.4 ± 0.1	0.1 ± 0	55 ± 12	0.4 ± 0.1
	A411	0.2 ± 0	0.1 ± 0	65 ± 14	0.4 ± 0.1
	A413	0.2 ± 0	0.1 ± 0	75 ± 0	0.4 ± 0.1
	A414	0.2 ± 0	0.1 ± 0	55 ± 12	0.2 ± 0.2
	A801	0.2 ± 0	0.2 ± 0.1	85 ± 14	0.2 ± 0.2
	A814-a	0.2 ± 0	0.2 ± 0	50 ± 0	0.4 ± 0.2
	A824-a	0.2 ± 0	0.1 ± 0	55 ± 12	0.4 ± 0.2
PH Hospital	B107-a	0.4 ± 0.1	0.2 ± 0.1	50 ± 0	0.4 ± 0.2
	B425-a	0.4 ± 0.1	0.2 ± 0.1	65 ± 14	0.4 ± 0.1
	B425-b	0.4 ± 0.1	0.1 ± 0	50 ± 0	0.4 ± 0.1
	B426-a	0.2 ± 0.1	0.1 ± 0	50 ± 0	0.4 ± 0.2
	B475-a	0.4 ± 0.1	0.2 ± 0	65 ± 14	0.4 ± 0.1
	B506-a	0.2 ± 0	0.2 ± 0	55 ± 12	0.2 ± 0.1
	B705-b	0.2 ± 0	0.2 ± 0	60 ± 14	0.2 ± 0.1
JU Hospital	C604	0.2 ± 0	0.2 ± 0	55 ± 12	0.4 ± 0.1
	C705	0.4 ± 0.1	0.1 ± 0	50 ± 0	0.4 ± 0.1
	C715-b	0.2 ± 0	0.2 ± 0	75 ± 0	0.4 ± 0.1
	C906	0.2 ± 0	0.1 ± 0	55 ± 12	0.2 ± 0.1
	*E. coli* ATCC	0.2 ± 0	0.1 ± 0	25 ± 0	0.4 ± 0.1
	*E. coli* Nissle	0.2 ± 0.1	0.2 ± 0.1	50 ± 0	0.4 ± 0.1
In- use concentration of the biocides		6[22]	4–4.8*	390–710[22]	2.5–5[22]

*Calculated from manufacturer’s instructions for use

## Discussion

The pattern of antibiotic resistance detected in the studied isolates is in line with the resistance encountered in different strains of *E. coli* isolated from various clinical or environmental sources worldwide [14, 15]. In the last few years it has been observed that *E. coli* is exhibiting resistance to more antibiotic classes, hence rendering these drugs ineffective in treating its infections. On the other hand, the high prevalence of *bla*CTXM 1 gene is expected since this gene was found to be the most prevalent ESBL enzyme producing genes in Jordan, particularly in *E. coli* [16, 17].

The results showing the prevalence of MDR and ESBL producing *E. coli* in the environment are alarming since they reveal the dissemination of ESBL bacteria not only in hospital environment, but also in the community. The spread of ESBL-producing microorganisms is of major concern to health organizations worldwide. In 2013, the CDC published a report listing the top 18 drug resistant threats to the United States [18]. ESBL-producing *Enterobacteriaceae* were within the group categorized as *“serious threat”*. Moreover, the spread of MDR organisms has caused the WHO to issue a recent report classifying ESBL-producing *Enterobacteriaceae* as a *“critical priority”*, where effective treatment is urgently required [19]. Infections with ESBL-producing bacteria leave limited choices in antibiotic treatment, where carbapenems are the only approved drugs of choice. This effectiveness was observed in this study where all isolates were susceptible to imipenem (Fig. 1).

The biocides investigated in this study were chosen from different classes; alcohols (ethanol), quaternary ammonium compounds (cetrimide), phenolics (chloroxylenol: 4-Chloro-3,5-xylenol,) and halogens (iodine). These classes are used extensively by health care sectors and /or household settings.

A large difference between the MIC and the in-use concentrations of the studied biocides was seen. This suggests that these biocides are effective against the isolated *E. coli* strains, whether MDR or non-MDR. Moreover, the MICs of household isolates and hospital isolates were comparable for all the biocides tested. They were also comparable to the quality control strain and the non pathogenic strain. These findings indicate that the extensive use of biocides in hospitals didn’t increase the MIC values of biocides, i.e. they didn’t have an impact on the resistance of *E. coli* isolates. Furthermore, we investigated the possible association of MDR with the MIC values of the isolates. No significant difference or association was found (*p* > 0.05).

The occurrence of potential cross-resistance between antimicrobial agents and antibiotics is still not well understood. Some reports have shown a relationship between biocide resistance and antibiotic resistance whilst others have failed to do so [2, 20, 21]. Cole et al. [20], performed a study on 1238 (Gram-positive and Gram-negative) bacterial isolates taken from different surfaces and locations from 60 houses. They didn’t observe any cross resistance between antibiotics and biocides. On the contrary, Moken et al. [21] reported cross-resistance between pine oil disinfectant and MDR. In their study, the cross resistance was thought to be through over-expression of multiple antibiotic resistance (*marA*) gene. Other studies have reported the induction of some resistant mechanisms, such as over-expression of efflux pumps or a decrease in growth rates and alteration in gene expression [2]. These are believed to be part of the bacterial stress response. The scientific controversy about the presence of cross-resistance with antibiotics has led the Scientific Committee on Emerging and Newly Identified Health Risks / Directorate General for Health and Consumers in the European Commission (Directorate General for Health and Consumers, 2009) to adopt and issue an opinion about *“Assessment of the Antibiotic Resistance Effects of Biocides”* in 2009 [2]. In this report, they state that *“there is convincing evidence that common mechanisms that confer resistance to biocides and antibiotics are present in bacteria”.* However, due to the limitations in identifying and characterizing cross-resistance in the targeted environment (in situ), the report concluded that more research is needed in this field.

## Conclusion

*E. coli* isolates from household and hospital environments showed high resistance rates to different classes of antibiotics without any significant differences between the two environments. For both groups, many *E. coli* isolates showed antibiotic multiple resistant patterns. ESBL-producing isolates were detected in both environments. *E. coli* isolates from both environments showed comparable MIC values of four of the widely used biocides, although the use of biocides in hospitals is more extensive than in households. Data generated from this study failed to show an association between antibiotic resistance and biocide resistance in *E. coli* isolates.
